# Discrepancies in symptom concerns and burden from the perspectives of parkinson's disease patients, caregivers, and physicians

**DOI:** 10.3389/fneur.2026.1794132

**Published:** 2026-05-04

**Authors:** Shohei Okusa, Toshiki Tezuka, Tomonori Nukariya, Yoshihiro Nihei, Jin Nakahara, Morinobu Seki

**Affiliations:** 1 Department of Neurology, Keio University School of Medicine, Tokyo, Japan; 2 Parkinson's Disease Center, Keio University Hospital, Tokyo, Japan; 3 Center for Parkinson's Disease Research, Keio University, Tokyo, Japan

**Keywords:** burden, caregiver, motor complication, motor symptom, non motor symptoms, Parkinson's disease, Patient, physician

## Abstract

**Background:**

Discrepancies in symptom concerns and the perceived burden among individuals with Parkinson's disease (PD), their caregivers, and their physicians can hinder effective care.

**Objectives:**

We investigated differences in symptom recognition and perceived impact among these groups.

**Participants and Methods:**

We conducted a cross-sectional study involving 193 PD patients, 80 caregivers, and their attending physicians at Keio University Hospital. All independently selected and ranked the three most troublesome symptoms from a comprehensive symptom list. The patients and physicians completed a questionnaire on motor complications including wearing-off, dyskinesia, delayed-on, no-on, and on-off, assessing both their recognition and the perceived burden.

**Results:**

Bradykinesia consistently emerged as the most concerning symptom in all three groups. Discrepancies in other symptoms' prioritization were observed. Tremor was more frequently prioritized by patients than by caregivers or physicians. The caregivers tended to emphasize symptoms that directly impacted caregiving, e.g., axial symptoms and non-motor manifestations such as urinary urgency and excessive daytime sleepiness. Motor complications (e.g., wearing-off, dyskinesia) were not highly prioritized by patients, even among those with longer disease durations. The physicians recognized these complications slightly more frequently and rated them as more distressing. Other motor complications, e.g., delayed-on, no-on, and on-off phenomena were more frequently recognized by patients than by their physicians.

**Conclusion:**

Significant perceptual gaps exist in the recognition and perceived burden of individual symptoms among PD patients, caregivers, and physicians. Improving communication, raising awareness of under-recognized symptoms, and fostering shared decision-making are essential for optimizing PD patients' individualized care and enhancing quality of life.

## 1 Introduction

Parkinson's disease (PD) is a neurodegenerative disorder characterized by dopaminergic neuron loss in the substantia nigra. Its cardinal motor symptoms progressively worsen and significantly impair quality of life (QOL). Individuals with PD also frequently experience non-motor symptoms, including autonomic dysfunction, sleep disturbance, psychiatric and cognitive impairments, and sensory abnormalities (1). The severity of these symptoms correlates with reduced QOL (2), and several studies suggest that non-motor symptoms may exert a greater impact on QOL than motor symptoms (3, 4). Motor complications such as wearing-off, dyskinesia, delayed-on, no-on, and on-off phenomena often develop as PD progresses. A study of patient-reported data identified such fluctuations as the most burdensome symptoms in advanced PD (5).

These motor, non-motor, and treatment-related complications all significantly contribute to QOL in PD, but their perceived severity and clinical burden vary among individuals. Several investigations documented mismatches in the perception of symptoms and treatment priorities between PD patients and physicians (6–9), extending beyond motor symptoms to non-motor symptoms and treatment satisfaction (6, 7). Even when PD patients and physicians recognize the presence of motor complications, their perceptions of how burdensome these complications are can differ (8, 10, 11), possibly affecting clinical decision-making.

Caregiver perspectives are also crucial, as symptoms in advanced or elderly PD patients often impose significant physical and psychological burdens on caregivers (12). Caregivers often highlight symptoms such as hallucinations, urinary dysfunction, and excessive daytime sleepiness (13). Their perspectives, however, are often underrepresented in clinical evaluations and research. With the aging PD population (14), incorporating caregivers‘ viewpoint has become increasingly essential to ensure patient-centered and sustainable care. Optimizing treatment strategies requires integrating the views of patients, caregivers, and physicians. Although discrepancies between patients and physicians, and between patients and caregivers, have been explored (6–10, 13, 15, 16), few studies have systematically compared all three perspectives. Qualitative assessments of these groups' viewpoints are available on topics such as disease stage, activity level, and motor fluctuations (17–19), highlighting the need for more comprehensive, quantitative approaches in real-world settings.

This study aimed to examine differences in symptom-related concerns and perceived burden among PD patients, caregivers, and attending physicians, with a focus on patient–physician discrepancies in motor complication perception. By comparing responses, we sought to identify perceptual gaps and obtain insights to improve comprehensive PD management.

## 2 Subjects and methods

### 2.1 Subjects

This cross-sectional study included 200 consecutive PD patients attending an outpatient clinic at Keio University Hospital and 86 of their caregivers agreed to participate between February and June 2023. In addition to patient questionnaires, caregiver questionnaires were collected when an accompanying family member or other care partner who was familiar with the patient's daily condition was present at the outpatient visit and agreed to participate. For the purpose of this study, caregiver respondents were defined pragmatically as accompanying individuals able to report on the patient's daily symptoms and care needs; therefore, they were not limited to spouses and were not necessarily the patient's sole primary caregiver. PD diagnosis was based on the International Parkinson and Movement Disorder Society (MDS) diagnostic criteria for clinically established or probable PD (20). Patients were stratified by disease durations into short-duration (< 6 years) and long-duration (≥6 years) groups, following Politis et al. (5) and by age at onset. Patients whose age at onset was 75 years or older were classified as the very-late-onset group, based on the proposal of the Japan Gerontological Society and the Japan Geriatrics Society redefining older adults as those aged 75 years or older. Because no universally accepted cutoff exists for a middle-onset PD subgroup, we prespecified patients with onset at 41–60 years as a pragmatic reference group representing typical non-elderly onset, centered around the usual age at onset of PD. Patients with onset at 61–74 years were not included in the the primary age-at-onset subgroup comparison in order to clarify differences associated with age at onset by comparing two more distinct groups. To explore the impact of age at onset, patients with disease duration < 10 years were stratified by age at onset. For the primary comparison, we focused on two more distinct groups—middle-onset (41–60 years) and very-late-onset (≥75 years)—to clarify differences associated with age at onset. In addition, the corresponding results for patients with onset at 61–74 years are provided in the Supplementary Materials. Caregiver burden was assessed using the Zarit Burden Interview (ZBI), with scores ≥21 indicating high burden, as previously described (21). The study was approved by the Keio University School of Medicine Ethics Committee (approval no. 20180052). Written consent was obtained from all participants.

### 2.2 Questionnaire

The questionnaire was developed with reference to the symptom framework reported by Politis et al., (5) but was modified for the purposes of the present study. Rather than using the original symptom list unchanged, we employed a more comprehensive set of symptom items to capture a broad range of motor and non-motor problems relevant to routine PD care. The questionnaire included items across multiple domains, including motor, autonomic, sleep, psychogenic/cognitive, sensory, and miscellaneous symptoms. To facilitate understanding, technical symptom terms were presented to patients and caregivers using simple, non-technical expressions in Japanese. For example, bradykinesia was described as “slowness of movement,” tremor as “shaking,” and rigidity as “muscle stiffness,” so that respondents could select symptoms based on their practical meaning rather than medical terminology. These modifications were made to allow a broader assessment of discrepancies in symptom prioritization among patients, caregivers, and physicians. This study included all physicians specializing in movement disorders at Keio University Hospital (*n* = 4). Patients selected symptoms impacting daily life, physicians identified those considered clinically significant, and caregivers ranked those affecting caregiving. To minimize bias, each completed the questionnaires separately without mutual disclosure. In addition, the patients and their physicians independently completed a study-specific questionnaire regarding motor complications. The questionnaire assessed whether wearing-off, dyskinesia, delayed-on, no-on, and on-off phenomena were present (yes/no). For each endorsed complication, perceived burden was rated on a 4-point scale: not troublesome, slightly troublesome, moderately troublesome, or very troublesome. PD patients, their attending physicians, and caregivers independently ranked the top three most bothersome symptoms. Rankings were weighted (3 points for first, 2 for second, 1 for third), following Politis et al. (5), but using a predefined comprehensive symptom list to standardize comparisons. For ranking purposes, symptoms with higher total scores were given higher positions.

The patients and their physicians also completed a questionnaire regarding motor complications, assessing the presence of wearing-off, dyskinesia, delayed-on, no-on, and on-off phenomena. Distress was rated on a four-point Likert scale. The PD patients' QOL was assessed with the Parkinson's Disease Questionnaire-8 (PDQ-8), and caregiver burden with the ZBI.

### 2.3 Statistical analyses

An *a priori* power analysis assuming a two-sided α of 0.05, a power of 0.90, and a moderate effect size (Cohen's *d* = 0.50) indicated that at least 172 participants were required. Demographics are presented as frequencies and means (±standard deviation). We used the χ^2^-test to analyze sex, initial symptoms, and response rates of concerns stratified by caregiver burden. The Mann-Whitney U-test was used for two-group comparisons. The level of statistical significance was set at *p* < 0.05. Commercial software (SPSS 29.0) was used for the analyses.

## 3 Results

### 3.1 The patients' and caregivers' demographic characteristics

All consecutive patients meeting the eligibility criteria during the study period were included. Based on the MDS diagnostic criteria for clinically established or probable PD (20), two patients did not meet the inclusion criteria. Five PD patients and six caregivers provided incomplete questionnaire responses and were also excluded. Caregiver questionnaires were obtained from 86 respondents, of whom 80 were included in the final analysis after exclusion of incomplete responses. The final analysis included data from 193 PD patients, their corresponding physicians, and 80 caregivers. Table 1 summarizes the patients‘ and caregivers' demographics. The final sample size of 193 participants, which exceeded the required number based on the power analysis.

**Table 1:** The clinical characteristics of the Parkinson's disease (PD) patients and caregivers of PD patients.

PD patients (*n* = 193)	
Male : female, *n*	106 : 87
Age, yrs	72.6 ± 8.8 (27–92)
Onset age, yrs	63.6 ± 11.0 (9–86)
Duration, yrs	9.0 ± 5.8 (1–27)
Length of treatment, yrs	7.4 ± 5.4 (1–27)
Hoehn & Yahr stage	2.9 ± 0.8
Initial symptom	Tremor 92 (47.7) Gait disturbance 48 (24.9) Bradykinesia 47 (24.4) Others 6 (3.1)
PDQ-8 score, points	7.7 ± 5.2
Caregivers (*n* = 80)	
Age, yrs	67.1 ± 11.0 (34–91)
Relationship with patient	Spouse 54 (67.5) Child 18 (22.5) Siblings 4 (5.0) Other 4 (5.0)
ZBI score, points	20.0 ± 14.5

The data are mean heSD (range) or n (%). PDQ-8, Parkinson's Disease Questionnaire, ZBI, Zarit care burden interview.

### 3.2 Comparative ranking of symptom concerns among the patients, physicians, and caregivers

Table 2 presents the rankings of symptom concerns in the groups of patients, physicians, and caregivers, respectively. Bradykinesia was the top concern across all groups. Gait disturbances, falls, and postural abnormality also commonly ranked in the top 10 of symptom concerns across groups. Tremor ranked second in the patient group, whereas it ranked fourth in the physician group, and it didn't rank in the top 10 in the caregiver group.

**Table 2:** Comparative rankings of symptom concerns from the perspectives of all participants.

Rank	PD (n = 193)	Total score	1^st^ choice	2nd choice	3rd choice	Three-choice complaint prevalence	Attending physician	Total score	1st choice	2^nd^ choice	3rd choice	Three-choice complaint prevalence	Caregiver (n = 80)	Total score	1st choice	2nd choice	3rd choice	Three-choice complaint prevalence
1	Bradykinesia	161	14.0	17.1	7.3	38.3	Bradykinesia	224	18.1	20.2	21.2	59.5	Bradykinesia	76	18.8	16.3	6.3	41.4
2	Tremor	123	14.0	8.8	4.1	26.9	Gait disturbance	176	17.1	14.5	10.9	42.5	Falls	40	10.0	6.3	7.5	23.8
3	Posture abnormality	99	9.8	8.3	5.2	23.3	Wearing off	128	16.1	6.2	5.7	28	Postural instability	33	6.3	8.8	5.0	20.1
4	Postural instability	80	5.2	9.3	7.3	21.8	Tremor	95	10.4	6.2	5.7	22.3	Postural abnormality	29	3.8	10.0	5.0	18.8
5	Constipation	74	4.7	8.8	6.7	20.2	Postural abnormality	80	8.3	6.7	3.1	18.1	Wearing off	27	6.3	5.0	5.0	16.3
6	Rigidity	70	7.3	4.7	5.2	17.1	Falls	71	7.8	5.2	3.1	16.1	Frequent urination	27	5.0	7.5	3.8	16.3
7	FOG	64	6.2	5.2	4.1	15.5	Rigidity	63	4.1	6.2	7.8	18.1	FOG	23	6.3	5.0	0	11.3
8	Gait disturbance	55	6.2	3.1	3.6	13.0	Pain	55	4.1	3.6	8.8	16.5	EDS	23	5.0	5.0	3.8	13.8
9	Falls	51	4.7	4.7	3.1	12.4	FOG	36	3.1	3.6	2.1	8.8	Constipation	22	5.0	3.8	5.0	13.8
10	Fatigue	45	4.1	4.1	2.6	10.9	Dyskinesia	33	0.5	6.2	3.1	9.8	Gait disturbance	20	5.0	2.5	5.0	12.5

The data are percentages. EDS, excessive daytime sleepiness; FOG, freezing of gait.

Concerning non-motor symptoms, there were notable differences among the groups. Constipation and fatigue ranked in the top 10 in the patient group, while pain ranked in the physician group. In the caregiver group, frequent urination, excessive daytime sleepiness, and constipation were in the top 10 of symptom concerns. Regarding motor complications, wearing-off ranked in the top 10 in both the physician and caregiver groups, whereas it didn't rank in the patient group.

### 3.3 Agreement between the patients and physicians on the most bothersome symptoms

We assessed whether physicians identified the same primary concerns as their patients (Table 3, Supplementary Figure S1). Physicians generally showed higher agreement with patients on motor symptoms than non-motor symptoms. However, agreement was relatively lower for tremor, postural instability/falls, dysarthria, dysphagia, and sialorrhea. Among non-motor symptoms, physicians demonstrated relatively better agreement with patients regarding orthostatic hypotension, depression, pain, and diplopia.

**Table 3:** Agreement between the patients and physicians on individual symptoms identified as most bothersome.

Category	Patients, n^*^	Concordance rate, %^**^	Category	Patients, n^*^	Concordance rate, %^**^
Motor:			Psychogenic/Cognitive		
Bradykinesia	74	68.9	Depression	6	66.7
Tremor	52	40.4	Hallucination	6	33.3
Rigidity	33	48.5	Delusion	0	–
Wearing off	16	75.0	Anxiety	3	0
Dyskinesia	16	81.3	Cognitive decline	7	28.6
Postural instability	62	40.3	Apathy	0	–
Postural abnormality	45	62.2	Poor concentration	8	12.5
Gait disturbance	50	74.0	Panic	1	0
Dysarthria/dysphagia/sialorrhea	19	15.8	Impulse control disorder	0	–
Autonomic:			Sensory:		
Constipation	41	24.4	Olfactory disorder	4	0
Nausea/vomiting	0	–	Taste disorder	0	–
Frequent urination	27	14.8	Pain	7	42.9
Loss of libido	1	0	15-2.4,-1.5237ptNumbness	1	0
Orthostatic hypotension	7	42.9	Miscellaneous:		
15-7.4,-1.5261ptAbnormal sweating	5	20.0	Diplopia	5	40.0
Sleep:			Weight loss	2	0
Insomnia	11	36.4	Edema	7	14.3
Daytime sleepiness	18	27.8	Dyspnea	2	0
REM sleep behavior disorder	4	25.0	Fatigue	21	33.3
Restless leg syndrome	2	0	–	–	–

^*^No. of patients who selected the symptoms as bothersome.

^**^Percentage of patients for whom the attending physician selected the same symptoms as bothersome.

### 3.4 Impact of disease duration on symptom concerns among the patients, physicians, and caregivers

To evaluate the impact of disease duration, we compared symptom concerns between the short-duration (*n* = 67) and long-duration (*n* = 126) groups (Supplementary Tables S1, S2). Patients, physicians, and caregivers all identified bradykinesia as the most frequent concern in both groups. Patients consistently ranked tremor higher than physicians or caregivers, regardless of disease duration.

Among non-motor symptoms, patients with long disease duration most frequently reported constipation. Caregivers in the short-duration group also emphasized constipation, whereas caregivers in the long-duration group more often cited urinary frequency. Physicians and patients, however, showed low overall agreement on non-motor symptoms. Notably, even patients with long disease duration did not rank motor complications such as wearing-off and dyskinesia among their top concerns.

### 3.5 Impact of age at onset on symptom concerns among patients, physicians, and caregivers

To explore the impact of age at onset, we examined symptom concerns among patients with disease duration < 10 years according to age at onset. For the primary comparison, we focused on middle-onset (41–60 years) and very-late-onset (≥75 years) groups to better highlight differences between more distinct age-at-onset categories; the corresponding results for the 61–74-year group are provided in the Supplementary Materials (Supplementary Tables S3, S4). Patients in the very late-onset group were older and had higher Hoehn & Yahr (HY) stages. In the middle-onset group, physicians most frequently ranked wearing-off instead of bradykinesia, whereas patients did not include wearing-off among their top five concerns. Middle-onset patients more often reported freezing of gait, whereas very-late-onset patients more commonly reported vulnerability to falling. These findings suggest that the age at onset may influences symptom prioritization by both patients and physicians.

### 3.6 Recognition gaps in motor complications between the patients and their physicians

Motor complications, including wearing-off and dyskinesia, are significant issues in advanced PD. To clarify their clinical perception, we examined discrepancies between patients and their attending physicians in both the recognition and perceived burden of motor complications (Table 4). Physicians recognized wearing-off and dyskinesia slightly more frequently than patients. However, physicians and patients showed a greater discrepancy in how they rated the burden of these complications. Physicians rated these complications as more distressing compared to the patients. Patients more frequently recognized other motor complications (e.g., delayed-on, no-on, and on-off phenomena) than their physicians, and the two groups showed the low concordance regarding the presence of these symptoms. The perceived burden of these complications differed depending on the complication type: physicians rated delayed-on as more burdensome, while patients considered no-on more distressing.

**Table 4:** Recognition and perceived burden of motor complications: comparison between the patients and their physicians.

Total (*n* = 193)	Wearing-off	Dyskinesia	Delayed-on	No-on	On-off
Reported by physician	41.5	24.9	16.1	13.0	9.8
Reported by patient	39.4	19.7	23.8	28.0	21.8
Concordance rate	57.6	53.6	37.5	27.4	29.8
Severity concordance rate	20.2	33.3	52.4	58.8	14.3
Burden: patient > physician^*^	26.3	16.7	19.0	23.5	42.9
Burden: physician > patient^**^	53.5	50.0	28.6	17.6	42.8
Short duration (*n* = 67)					
Reported by physician	16.4	6.0	7.5	4.5	3.0
Reported by patient	23.9	9.0	16.4	17.9	7.5
Concordance rate	9.1	25.0	33.3	15.4	16.7
Severity concordance rate	0	0	0	50	0
Severity (patient > physician)	22.2	0	50	50	100
Severity (physician > patient)	77.8	100	50	0	0
Long duration (*n* = 126)					
Reported by physician	54.8	34.9	20.6	17.5	13.5
Reported by patient	47.6	25.4	27.8	33.3	29.4
Concordance rate	59.3	58.3	38.6	30.6	31.7
Severity concordance rate	41.7	35.7	64.7	60.0	15.4
Severity (patient > physician)	10.4	17.9	11.8	20.0	38.5
Severity (physician > patient)	47.9	46.4	23.5	20.0	46.2

^*^Patients rated as more distressing.

^**^Physicians rated as more distressing.

### 3.7 Patient characteristics and symptom concerns in relation to the caregiver burden

We investigated whether patient-reported concerns differed according to the level of caregiver burden (Supplementary Table S5). Patients in the high-burden caregiver group were significantly older, had more advanced HY stages, and showed higher PDQ-8 scores, indicating poorer quality of life. Patients in this group reported tremor onset significantly less frequently. Although no symptom reached statistical significance, patients with high caregiver burden more frequently reported dysphagia (*p* = 0.07) and psychiatric symptoms (*p* = 0.06).

## 4 Discussion

We investigated differences in symptom concerns and perceived burden among PD patients, physicians, and caregivers. All groups consistently identified bradykinesia as the primary concern. In contrast, patients appeared to place less emphasis on relatively predictable motor complications such as wearing-off and dyskinesia in their overall ranking of symptom concerns, while still recognizing specific fluctuation phenomena, including delayed-on, no-on, and on-off episodes. Physicians underappreciated tremor despite its impact on patients, whereas recognition and prioritization of non-motor symptoms varied widely across groups. The caregiver burden was closely associated with disease severity and symptom complexity.

### 4.1 Motor symptoms

Bradykinesia, a mandatory criterion in the PD diagnostic criteria (20), was consistently identified as the most concerning symptom across all groups, underscoring its central role in both the diagnosis and daily burden of PD. This consistency highlights bradykinesia's functional impact on essential motor activities. As an exception, physicians managing middle-onset PD patients most frequently selected wearing-off as their top concern (Supplementary Table S4), rather than bradykinesia. This difference may reflect increased clinical attention to motor fluctuations which commonly emerge in this age group.

Patients prioritized tremor as a major concern, while physicians and caregivers rated it lower, showing a marked perceptual gap. Although physicians often consider tremor less disabling than bradykinesia or axial symptoms, tremor is often resistant to dopaminergic therapy and can significantly impair patients' physical and social QOL (22, 23). Therapeutic options for troublesome tremor may also be limited in practice. Although anticholinergics can sometimes be considered, their antitremor effect is generally less than that of levodopa, and their clinical use is usually restricted to selected younger and cognitively intact patients because of adverse effects, including cognitive impairment, dry mouth, constipation, and urinary retention (24, 25). This finding aligns with a recent report by Mammen et al. in which patients frequently described tremor as distressing, while family members rarely mentioned it as a primary concern (15). This underrecognition may reflect clinical underestimation of tremor's functional and emotional impacts rather than a simple difference in perceived importance. Interestingly, tremor onset was more common in patients whose caregivers reported lower burden, suggesting that while tremor distresses patients, it imposes less direct caregiving demand than symptoms requiring physical assistance or supervision.

All groups consistently prioritized axial symptoms, including postural abnormalities, gait disturbances, falls, and freezing of gait. These symptoms are typically less responsive to dopaminergic treatment and are strongly associated with loss of independence (26–28). Therapeutic options for these symptoms remain particularly challenging. Beyond optimization of dopaminergic therapy, pharmacologic strategies for levodopa-resistant gait, balance, and postural symptoms remain limited, and evidence for alternative approaches, including cholinergic therapies such as rivastigmine, has been mixed or inconclusive (29). Therefore, non-pharmacologic strategies, including rehabilitation, gait and balance training, and multidisciplinary supportive care, should be emphasized in clinical practice (30, 31). Caregivers‘ prioritization of these symptoms likely reflects their direct impact on mobility, the need for supervision, and injury prevention, thereby contributing significantly to caregivers' stress.

### 4.2 Non-motor symptoms

Physicians often underrecognize non-motor symptoms, whereas patients and caregivers perceive them as challenging to manage (32). Our analyses revealed considerable variation in how each group perceived and prioritized non-motor symptoms. Although non-motor symptoms are well-established contributors to reduced QOL in PD (2–4, 23), the consensus on the most bothersome symptoms was lacking. Patients frequently ranked constipation and fatigue, while physicians more often emphasized pain. One possible explanation is that pain may be more readily recognized during clinical encounters because it is often explicitly expressed and may prompt more immediate therapeutic consideration, including medication adjustment, analgesic treatment, rehabilitation, or referral to other specialties. By contrast, symptoms such as fatigue and constipation are often chronic, multifactorial, and less systematically explored during routine consultations. This discrepancy has important clinical implications. When physicians prioritize symptoms that appear more clinically actionable, patient concerns that are highly burdensome but less readily treatable may remain underrecognized. Such mismatches may influence symptom recognition, shape clinical decision-making toward more readily manageable symptoms, and ultimately hinder truly patient-centered care. The low concordance between patients and physicians regarding symptoms such as fatigue and constipation therefore underscores the importance of structured symptom assessment and active communication during routine consultations.

The caregivers often prioritized symptoms that directly affected caregiving tasks, e.g., urinary frequency and excessive daytime sleepiness. This discrepancy in prioritization between patients and caregivers is consistent with the finding in a recent systematic review by Tosin et al. (16), reporting that while both patients and caregivers view non-motor symptoms as key QOL determinants, they tend to emphasize different non-motor symptoms. For example, the patients focused more on pain and fatigue, whereas the caregiver partners were more likely to highlight psychiatric symptoms (e.g., hallucinations and delusions). These differences reflect the distinct burdens and emphasize the need for individualized and comprehensive management of non-motor symptoms.

These findings reinforce the importance of structured, patient-centered assessments and improved communication to ensure comprehensive recognition of non-motor symptom burden in clinical decision-making.

### 4.3 Motor complications

We examined discrepancies between patients and their attending physicians in both the recognition and perceived burden of motor complications. Our findings suggest that patient perceptions differed according to the type of motor complication. In the overall ranking analysis, patients did not place relatively predictable motor complications, particularly wearing-off and dyskinesia, among their highest symptom concerns. In contrast, in the symptom-specific paired analysis, patients more frequently recognized other fluctuation phenomena, such as delayed-on, no-on, and on-off episodes, than their physicians, and the two groups showed overall low concordance.

This distinction may help explain the apparent inconsistency between the ranking and recognition analyses. Patients may be less likely to prioritize the broad clinical concepts of wearing-off or dyskinesia when asked to identify their most bothersome symptoms, yet still clearly perceive episodic and fluctuating treatment responses in daily life. In particular, unpredictable fluctuations may be especially troublesome from the patient perspective, as previous work has shown that both increased “on” time and greater predictability of “off” time are highly valued by people with PD (33).

Previous studies have described that patients show limited awareness of wearing-off and have difficulty accurately recognizing motor fluctuations (8, 11); potentially lead to underreporting or misrecognition. For example, Matthews et al. reported that although 87% of patients and 74% of caregiver partners believed they understood the concept, only 30 and 17%, respectively, could accurately define it (10). In our study, physicians recognized wearing-off and dyskinesia slightly more frequently than patients, although the recognition gap for wearing-off was relatively small (Table 4). In contrast, patients more frequently recognized delayed-on, no-on, and on-off phenomena than their physicians.

These findings suggest that patient recognition may differ according to how motor complications are experienced and framed. Patients may be less likely to identify broad clinical constructs such as wearing-off or motor complications when asked to rank their major concerns, while still recognizing concrete and episodic fluctuation phenomena in daily life. Thus, our findings do not necessarily contradict previous reports of limited awareness; rather, they suggest that awareness may be greater for symptom-specific or unpredictable experiences than for umbrella clinical terminology.

Although patients and physicians showed similar recognition rates for wearing-off, they differed markedly in their assessment of its burden. While physicians and caregivers prioritized wearing-off and dyskinesia are well-established contributors to disease burden in advanced PD (5, 11), our patients (even those with longer disease durations) did not rank motor complications as highly troublesome (Supplementary Table S2). By contrast, physicians prioritized them, and caregivers also identified wearing-off as burdensome. Physicians rated wearing-off and dyskinesia significantly more troubling than the patients did (Table 4). This may reflect the fact that physicians are particularly attentive to identifying motor fluctuations and troublesome dyskinesia, as these complications are often clinically actionable at this stage of the disease. In many cases, relatively simple therapeutic adjustments, such as modifying dopaminergic medications or adding adjunctive therapies, can be considered, while in selected patients, more advanced interventions such as device-aided therapies may also be proposed. The frequent reluctance of patients to accept neurologists' recommendations for device-aided therapies also supports this perspective, and a transition to a different treatment approach may require time and repeated discussion. By contrast, this discrepancy may also reflect patients' subjective experiences—many PD patients may prefer an “on” state with mild dyskinesia to an “off” state, aligning with the concept of “non-troublesome dyskinesia.” Even relatively mild wearing-off, however, may still interfere with daily function and quality of life.

Nevertheless, our patients did not rank wearing-off among their top concerns and rated its burden as lower than that perceived by their physicians. The patients emphasized levodopa-resistant symptoms (e.g., tremor, postural abnormalities, and gait disturbances) which are persistent and less responsive to medication. These findings suggest that patients prioritize chronic, treatment-refractory motor symptoms over fluctuating complications like wearing-off, while physicians tend to emphasize medication-related fluctuations due to their modifiability. The relatively low prioritization of motor complications may not be explained solely by unfamiliarity with the terminology. Instead, patients may have been more likely to identify the specific symptoms they experienced during the off state, such as tremor, gait disturbance, or bradykinesia, rather than the broader concept of motor complications. Limited patient understanding of wearing-off may also contribute to this discrepancy, underscoring the need for education to improve symptom awareness (19).

Our cohort included a relatively large proportion of older patients compared to that of Politis et al. (5). Motor complications such as wearing-off and dyskinesia tend to be most troublesome in the earlier phases of the advanced stage, i.e. the middle stage (34). In later stages, persistent motor impairments may overshadow the functional impact of fluctuating symptoms (12), reducing their perceived burden.

Overall, our findings underscore the importance of evaluating the burden of motor complications from both clinical and patient-reported perspectives. Structured interviews and symptom questionnaires may facilitate recognition and communication of wearing-off in routine practice, while symptom diaries and digital monitoring may provide complementary information on the temporal pattern and variability of motor fluctuations between visits. These approaches may help improve the characterization of motor complications and support more individualized treatment decisions, although their impact on patient–physician perceptual alignment and clinical outcomes requires further study (35–37).

### 4.4 Caregiver burden

The caregiver burden is a critical aspect of PD management, particularly in the context of an aging population and the growing number of late-stage patients. Fabbri et al. noted that insufficient recognition and support for caregivers may lead to caregiver burnout and the emergence of the so-called ‘invisible patient' — a caregiver whose own wellbeing is overlooked in the focus on patient care (12). It has been demonstrated that longer disease duration, greater disease severity, pronounced negative emotions, and cognitive impairment are associated with an increased caregiver burden (38). In our study, patients whose caregivers reported a high burden were significantly older, had more advanced HY stages, and exhibited poorer QOL. These patients reported dysphagia and psychiatric symptoms more frequently, suggesting that functional impairments such as swallowing difficulties and neuropsychiatric manifestations may impose a disproportionate strain on caregivers.

Interestingly, patients in the high-burden group reported tremor onset significantly less often, possibly indicating that tremor alone contributes less to the caregiver workload. These findings underscore the importance of holistic care strategies that support both patients and caregivers, particularly in the context of aging and advanced disease. Longacre et al. emphasized that caregivers often perceive symptoms differently from patients and physicians, and their input can provide valuable insights into underreported issues such as fatigue, sleep disturbances, and hallucinations (13).

### 4.5 Clinical implications and future directions

Researchers have described the importance of integrating patient-reported concerns into PD care to align treatment with individual experiences and goals (39). For example, Nomoto et al. demonstrated a mismatch between physician-rated treatment success and patient satisfaction, especially when non-motor symptoms are insufficiently managed (6). Our findings highlight the importance of open and ongoing communication among patients, caregivers, and physicians to better align clinical priorities with patients' experiences and preferences in daily life, particularly when managing complex non-motor symptoms and motor fluctuations. In this context, approaches that support patient-centered care and incorporate the perspectives of both patients and caregivers may help facilitate more individualized care planning. Furthermore, clinicians must adopt a holistic approach to care that incorporates the diverse perspectives of patients, caregivers, and healthcare providers. This includes adequately addressing levodopa-resistant symptoms and the caregiver burden, both of which clinicians often underrecognized. By tailoring care to each patient's clinical context, clinicians can improve QOL and reduce caregiver stress.

A strength of this study is that it incorporated the perspectives of patients, caregivers, and attending physicians, allowing comparison of symptom recognition and prioritization across motor and non-motor domains. However, several study limitations should be noted; potential recall bias, limited generalizability due to the single-center design, and possible influence of the predefined symptom list on symptom interpretation and ranking. Furthermore, caregiver data were available for only a subset of patients. In this study, caregiver respondents were defined pragmatically as accompanying family members or other care partners who were familiar with the patient's daily condition, rather than as a uniformly defined primary caregiver population. Accordingly, the caregiver sample may have represented a self-selected subgroup of individuals who were present and willing to participate at the outpatient visit. This may have led to overrepresentation of more engaged caregivers and/or patients with greater care needs. In addition, patients with milder symptoms were often able to attend the hospital independently, whereas those accompanied by caregivers generally had more advanced disease and greater functional dependence. Because we did not directly compare patients with and without caregiver responses, the effect of this imbalance was not formally assessed. Therefore, the caregiver-related findings may not be fully representative of the broader caregiver population and should be interpreted with caution when considering their generalizability.

In conclusion, we identified significant discrepancies in the recognition and prioritization of PD symptoms among patients, caregivers, and physicians. Addressing these differences through improved communication, targeted education, and integrated care will be key to advancing individualized, patient-centered PD care.

## Data Availability

The datasets generated and/or analyzed during the study are available from the corresponding author upon reasonable request.
