# Supplementary material for: Discrepancies in symptom concerns and burden from the perspectives of parkinson's disease patients, caregivers, and physicians
Source: Front Neurol. 2026 May 4;17:1794132. doi: 10.3389/fneur.2026.1794132 (PMC13180621; doi:10.3389/fneur.2026.1794132)
Supplement: Supplementary file 1 [file Table_1.docx]

**Suppl. Table S1.** The clinical characteristics of the patients with Parkinson's disease (PD), stratified by their disease durations

|  | **Short-duration PD (<6 yrs)**  **(n=67)** | **Long-duration PD**  **(≥6 yrs)**  **(n=126)** | **p-value** |
| --- | --- | --- | --- |
| Age, yrs | 72.9 ± 7.9 | 72.4 ± 9.3 | 0.70 |
| Male : female | 38 : 29 | 68 : 58 | 0.76 |
| Hoehn & Yahr stage | 2.6 ± 0.7 | 3.1 ± 0.8 | <0.001 |
| Onset age, yrs | 69.3 ± 8.1 | 60.5 ± 11.1 | <0.001 |
| Duration, yrs | 3.5 ± 1.2 | 11.9 ± 5.2 | <0.001 |
| Length of treatment, yrs | 2.7 ± 1.3 | 9.9 ± 5.1 | <0.001 |

The data are n or mean ± SD.

**Suppl. Table S2.** Ranking of symptom concerns: Comparison by disease duration

|  | **Short-duration (<6 yrs)** | | | | | | **Long-duration (≥6 yrs)** | | | | | | |
| --- | --- | --- | --- | --- | --- | --- | --- | --- | --- | --- | --- | --- | --- |
| **Rank** | **PD patients**  **(n=67)** | **Total score** | **1st choice** | **2nd choice** | **3rd choice** | **Three-choice complaint prevalence** | **PD patients**  **(n=126)** | **Total score** | **1st choice** | **2nd choice** | **3rd choice** | **Three-choice complaint prevalence** |  |
| 1 | Bradykinesia | 52 | 16.4 | 11.9 | 4.5 | 32.8 | Bradykinesia | 109 | 12.7 | 19.8 | 8.7 | 41.2 |  |
| 2 | Tremor | 52 | 16.4 | 11.9 | 4.5 | 32.8 | Tremor | 71 | 12.7 | 7.1 | 4.0 | 23.8 |  |
| 3 | Postural abnormality | 41 | 11.9 | 10.4 | 4.5 | 26.8 | Postural abnormality | 58 | 8.7 | 7.1 | 5.6 | 21.4 |  |
| 4 | Postural instability | 34 | 6.0 | 11.9 | 9.0 | 26.9 | Freezing of gait | 51 | 7.9 | 6.3 | 4.0 | 18.2 |  |
| 5 | Rigidity | 30 | 10.4 | 4.5 | 4.5 | 19.4 | Constipation | 46 | 3.2 | 9.5 | 7.9 | 20.6 |  |
| **Rank** | **Physician** | **Total score** | **1st choice** | **2nd choice** | **3rd choice** | **Three-choice complaint prevalence** | **Physician** | **Total score** | **1st choice** | **2nd choice** | **3rd choice** | **Three-choice complaint prevalence** |  |
| 1 | Bradykinesia | 95 | 26.0 | 20.9 | 19.4 | 66.3 | Bradykinesia | 129 | 13.5 | 19.8 | 22.2 | 55.5 |  |
| 2 | Gait disturbance | 60 | 13.4 | 19.4 | 10.4 | 43.2 | Gait disturbance | 116 | 19.0 | 11.9 | 11.1 | 42.0 |  |
| 3 | Wearing off | 43 | 17.9 | 1.5 | 7.5 | 26.9 | Wearing off | 85 | 15.1 | 8.7 | 4.8 | 28.6 |  |
| 4 | Tremor | 33 | 13.4 | 13.4 | 4.5 | 31.3 | Tremor | 62 | 11.1 | 4.8 | 6.3 | 22.2 |  |
| 5 | Rigidity | 31 | 4.5 | 13.4 | 14.9 | 32.8 | Postural abnormality | 57 | 9.5 | 6.3 | 4.0 | 19.8 |  |
| **Rank** | **Caregiver**  **(n=25)** | **Total score** | **1st choice** | **2nd choice** | **3rd choice** | **Three-choice complaint prevalence** | **Caregiver**  **(n=55)** | **Total score** | **1st choice** | **2nd choice** | **3rd choice** | **Three-choice complaint prevalence** |  |
| 1 | Bradykinesia | 28 | 16.0 | 24.0 | 16.0 | 56.0 | Bradykinesia | 48 | 20.0 | 12.7 | 1.8 | 34.5 |  |
| 2 | Postural abnormality | 14 | 12.0 | 8.0 | 4.0 | 24.0 | Falls | 31 | 10.9 | 7.3 | 9.1 | 27.3 |  |
| 3 | Postural instability | 11 | 0 | 20.0 | 4.0 | 24.0 | Wearing off | 26 | 9.1 | 7.3 | 5.5 | 21.9 |  |
| 4 | Tremor | 10 | 12.0 | 0 | 4.0 | 16.0 | Postural instability | 22 | 9.1 | 3.6 | 5.5 | 18.2 |  |
| 5 | Constipation | 10 | 8.0 | 8.0 | 0 | 16.0 | Frequent urination | 20 | 5.5 | 7.3 | 5.5 | 18.3 |  |

The data are percentages.

**Suppl. Table S3.** Clinical characteristics of the patients with Parkinson's disease (PD), stratified by disease onset age

|  | **Middle-onset PD (n=22)** | **Very-late-onset PD (n=28)** | **p-value** |
| --- | --- | --- | --- |
| Age, yrs | 59.8 ± 6.1 | 83.0 ± 3.6 | <0.001 |
| Male : female | 14 : 8 | 17 : 11 | 0.80 |
| Hoehn & Yahr stage | 2.5 ± 0.5 | 3.0 ± 0.9 | 0.03 |
| Onset age, yrs | 53.2 ± 5.6 | 77.9 ± 3.5 | <0.001 |
| Duration, yrs | 6.5 ± 2.2 | 5.1 ± 2.5 | 0.08 |
| Length of treatment, yrs | 5.3 ± 2.4 | 4.0 ± 2.3 | 0.11 |
| Initial symptom, n | Tremor 7  Gait disturbance 2  Bradykinesia 13 | Tremor 14  Gait disturbance 13  Bradykinesia 1 | <0.001 |

The data are n or mean ± SD.

**Suppl. Table S4.** Ranking of symptom concerns: Comparison by disease onset age

|  | **Middle-onset PD patients (41–60 yrs old)** | | | | | | **Very-late-onset PD patients (≥75 yrs old)** | | | | | | |
| --- | --- | --- | --- | --- | --- | --- | --- | --- | --- | --- | --- | --- | --- |
| **Rank** | **PD patients**  **(n=22)** | **Total score** | **1st choice** | **2nd choice** | **3rd choice** | **Three-choice complaint prevalence** | **PD patients**  **(n=28)** | **Total score** | **1st choice** | **2nd choice** | **3rd choice** | **Three-choice complaint prevalence** |  |
| 1 | Bradykinesia | 21 | 18.1 | 13.6 | 13.6 | 45.3 | Bradykinesia | 24 | 21.4 | 10.7 | 0.0 | 32.1 |  |
| 2 | Tremor | 19 | 22.7 | 9.1 | 0 | 31.8 | Tremor | 20 | 7.1 | 25.0 | 0.0 | 32.1 |  |
| 3 | Rigidity | 17 | 18.1 | 9.1 | 4.5 | 31.7 | Postural abnormality | 17 | 10.7 | 14.3 | 0 | 25.0 |  |
| 4 | Postural abnormality | 13 | 9.1 | 13.6 | 4.5 | 27.2 | Rigidity | 14 | 10.7 | 3.6 | 10.7 | 25.0 |  |
| 5 | Freezing of gait | 9 | 9.1 | 4.5 | 4.5 | 18.1 | Falls | 12 | 10.7 | 3.6 | 3.6 | 17.9 |  |
| **Rank** | **Physician**  **(n=22)** | **Total score** | **1st choice** | **2nd choice** | **3rd choice** | **Three-choice complaint prevalence** | **Physician**  **(n=28)** | **Total score** | **1st choice** | **2nd choice** | **3rd choice** | **Three-choice complaint prevalence** |  |
| 1 | Wearing off | 32 | 27.3 | 27.3 | 9.1 | 63.7 | Bradykinesia | 34 | 21.4 | 17.9 | 21.4 | 60.7 |  |
| 2 | Bradykinesia | 26 | 18.2 | 22.7 | 18.2 | 59.1 | Gait disturbance | 30 | 14.3 | 28.6 | 7.1 | 50.0 |  |
| 3 | Gait disturbance | 15 | 13.6 | 4.5 | 18.2 | 36.3 | Tremor | 20 | 14.3 | 10.7 | 7.1 | 50.0 |  |
| 4 | Falls | 9 | 9.1 | 4.5 | 4.5 | 18.1 | Falls | 20 | 14.3 | 10.7 | 7.1 | 32.1 |  |
| 5 | Rigidity | 8 | 9.1 | 4.5 | 0 | 13.6 | Rigidity | 9 | 3.6 | 3.6 | 14.3 | 21.5 |  |
| **Rank** | **Caregiver**  **(n=3)** | **Total score** | **1st choice** | **2nd choice** | **3rd choice** | **Three-choice complaint prevalence** | **Caregiver**  **(n=14)** | **Total score** | **1st choice** | **2nd choice** | **3rd choice** | **Three-choice complaint prevalence** |  |
| 1 | Bradykinesia | 6 | 33.3 | 33.3 | 33.3 | 100 | Bradykinesia | 16 | 28.6 | 14.3 | 0 | 42.9 |  |
| 2 | Postural instability | 4 | 0 | 66.7 | 0 | 66.7 | Falls | 12 | 14.3 | 14.3 | 14.3 | 42.9 |  |
| 3 | Postural abnormality | 3 | 33.3 | 0 | 0 | 33.3 | Postural instability | 8 | 0 | 21.4 | 14.3 | 35.7 |  |
| 3 | Constipation | 3 | 33.3 | 0 | 0 | 33.3 | Daytime sleepiness | 7 | 7.1 | 7.1 | 14.3 | 28.5 |  |
| – | – | – | – | – | – | – | Frequent urination | 7 | 7.1 | 14.3 | 0 | 21.4 |  |

|  | **Late-onset PD patients (61–74 yrs old)** | | | | | |
| --- | --- | --- | --- | --- | --- | --- |
| **Rank** | **PD patients**  **(n=77)** | **Total score** | **1st choice** | **2nd choice** | **3rd choice** | **Three-choice complaint prevalence** |
| 1 | Bradykinesia | 70 | 16.9 | 18.2 | 3.9 | 39.0 |
| 2 | Tremor | 55 | 18.2 | 5.2 | 6.5 | 29.9 |
| 3 | Constipation | 47 | 7.8 | 15.6 | 6.5 | 29.9 |
| 4 | Postural instability | 36 | 5.2 | 13.0 | 5.2 | 23.4 |
| 5 | Postural abnormality | 36 | 9.1 | 6.5 | 6.5 | 22.1 |
| **Rank** | **Physician**  **(n=77)** | **Total score** | **1st choice** | **2nd choice** | **3rd choice** | **Three-choice complaint prevalence** |
| 1 | Bradykinesia | 105 | 20.8 | 28.6 | 16.9 | 66.2 |
| 2 | Gait disturbance | 74 | 19.5 | 10.4 | 16.9 | 46.8 |
| 3 | Tremor | 54 | 16.9 | 7.8 | 3.9 | 28.6 |
| 4 | Postural abnormality | 33 | 9.1 | 6.5 | 2.6 | 18.2 |
| 5 | Pain | 31 | 5.2 | 9.1 | 6.5 | 20.8 |
| **Rank** | **Caregiver**  **(n=28)** | **Total score** | **1st choice** | **2nd choice** | **3rd choice** | **Three-choice complaint prevalence** |
| 1 | Bradykinesia | 32 | 17.9 | 25.0 | 10.7 | 53.6 |
| 2 | Tremor | 17 | 17.9 | 0 | 7.1 | 25.0 |
| 3 | Gait disturbance | 14 | 10.7 | 7.1 | 3.6 | 21.4 |
| 4 | Postural abnormality | 14 | 7.1 | 10.7 | 7.1 | 25.0 |
| 5 | Postural instability | 8 | 7.1 | 3.6 | 0 | 10.7 |

The data are percentages.

**Suppl. Table S5.** Comparison of patient and caregiver characteristics based on the caregiver burden

|  | **Low burden**  **n=51** | **High burden**  **n=29** | **p-value** |
| --- | --- | --- | --- |
| ZBI | 11.3 ± 5.3 | 35.2 ± 12.7 | <0.001 |
| Caregiver age | 66.1 ± 12.0 | 68.9 ± 8.8 | 0.40 |
| Patient age | 74.0 ± 7.5 | 77.3 ± 5.8 | 0.049 |
| Onset age | 63.8 ± 10.5 | 67.3 ± 10.9 | 0.14 |
| Disease duration | 10.1 ± 6.1 | 9.9 ± 6.5 | 0.84 |
| Treatment duration | 8.6 ± 6.1 | 8.2 ± 6.1 | 0.77 |
| Hoehn & Yahr stage | 3.2 ± 0.8 | 3.6 ± 0.7 | 0.03 |
| PDQ-8 score, points | 7.5 ± 5.1 | 10.3 ± 5.2 | 0.02 |
| Initial symptom, %: |  |  |  |
| Tremor | 51.0 | 27.6 | 0.04 |
| Bradykinesia | 19.6 | 24.1 | 0.63 |
| Gait disturbance | 25.5 | 44.8 | 0.08 |
| Another symptom | 3.9 | 3.4 | 0.70 |
| Symptom domains reported by patient, %: |  |  |  |
| Motor symptoms: | 94.1 | 93.1 | 0.60 |
| Motor complication | 25.5 | 10.3 | 0.10 |
| Postural abnormality/instability | 62.7 | 69.0 | 0.58 |
| Dysphagia and drooling | 11.8 | 27.6 | 0.07 |
| Autonomic symptoms: | 33.3 | 24.1 | 0.39 |
| Psychogenic/cognitive | 21.6 | 41.4 | 0.06 |
| Sleep-related | 25.5 | 20.7 | 0.63 |
| Sensory | 2.0 | 3.4 | 0.60 |
| Miscellaneous | 9.8 | 17.2 | 0.27 |

The data are mean ± SD or percentage. PDQ-8: Parkinson's Disease Questionnaire, ZBI: Zarit Care Burden Interview.
