# Supplementary figures and images for: Discrepancies in symptom concerns and burden from the perspectives of parkinson's disease patients, caregivers, and physicians
Source: Front Neurol. 2026 May 4;17:1794132. doi: 10.3389/fneur.2026.1794132 (PMC13180621; doi:10.3389/fneur.2026.1794132)

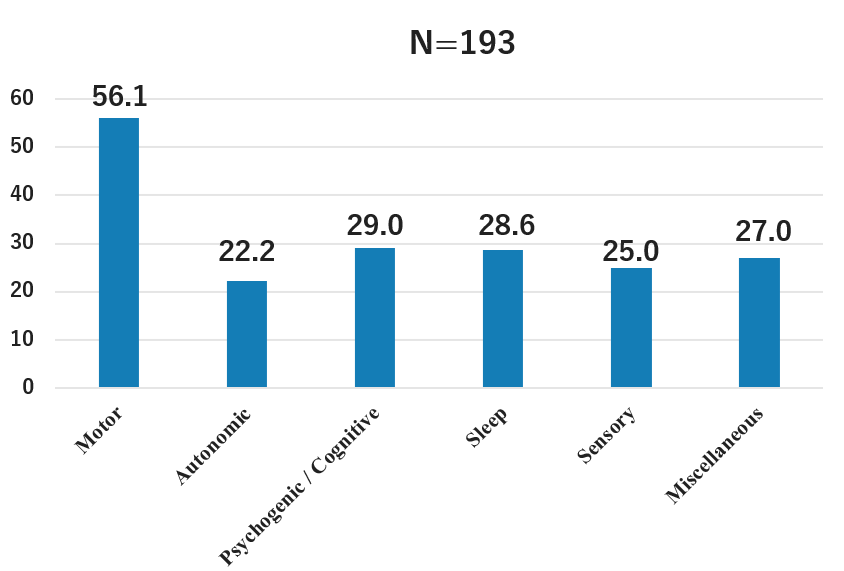

Supplement: Supplementary file 2 [file Image_1.png]
